# Lignan-Rich Sesame (*Sesamum indicum* L.) Cultivar Exhibits In Vitro Anti-Cholinesterase Activity, Anti-Neurotoxicity in Amyloid-β Induced SH-SY5Y Cells, and Produces an In Vivo Nootropic Effect in Scopolamine-Induced Memory Impaired Mice

**DOI:** 10.3390/antiox12051110

**Published:** 2023-05-17

**Authors:** Min-Young Kim, Sungup Kim, Jeongeun Lee, Jung-In Kim, Eunyoung Oh, Sang-Woo Kim, Eunsoo Lee, Kwang-Soo Cho, Choon-Song Kim, Myoung-Hee Lee

**Affiliations:** Department of Southern Area Crop Science, National Institute of Crop Science, Rural Development Administration, Milyang 50424, Republic of Korea; sesameup@korea.kr (S.K.); jel6418@korea.kr (J.L.); kji1204@korea.kr (J.-I.K.); lavondy10@korea.kr (E.O.); kimsw1021@korea.kr (S.-W.K.); awesomelee@korea.kr (E.L.); kscholove@korea.kr (K.-S.C.); kcs3925@korea.kr (C.-S.K.); emhee@korea.kr (M.-H.L.)

**Keywords:** sesame, lignan, amyloid-β, neurotoxicity, neuronal regeneration

## Abstract

Alzheimer’s disease, a major cause of dementia, is characterized by impaired cholinergic function, increased oxidative stress, and amyloid cascade induction. Sesame lignans have attracted considerable attention owing to their beneficial effects on brain health. This study investigated the neuroprotective potential of lignan-rich sesame cultivars. Among the 10 sesame varieties studied, Milyang 74 (M74) extracts exhibited the highest total lignan content (17.71 mg/g) and in vitro acetylcholinesterase (AChE) inhibitory activity (66.17%, 0.4 mg/mL). M74 extracts were the most effective in improving cell viability and inhibiting reactive oxygen species (ROS) and malondialdehyde (MDA) generation in amyloid-β_25-35_ fragment-treated SH-SY5Y cells. Thus, M74 was used to evaluate the nootropic effects of sesame extracts and oil on scopolamine (2 mg/kg)-induced memory impairment in mice compared to the control cultivar (Goenback). Pretreatment with the M74 extract (250 and 500 mg/kg) and oil (1 and 2 mL/kg) effectively improved memory disorder in mice (demonstrated by the passive avoidance test), inhibited AChE, and enhanced acetylcholine (Ach) levels. Moreover, immunohistochemistry and Western blot results showed that the M74 extract and oil reversed the scopolamine-induced increase in APP, BACE-1, and presenilin expression levels in the amyloid cascade and decreased BDNF and NGF expression levels in neuronal regeneration.

## 1. Introduction

The World Health Organization reported that approximately 50 million people worldwide have dementia [1]. Alzheimer’s disease (AD) is a major cause of dementia and is a growing global health challenge with significant implications for individuals and society [2]. AD is a common form of dementia that leads to memory impairment and cognitive dysfunction and mostly affects the elderly population aged >65 years. It is characterized by the deposition of senile plaques and neurofibrillary tangles (NFT) [3], neuronal death, and loss of synapses [4]. Pathogenic mechanisms underlying AD mostly include impaired cholinergic function, increased oxidative stress, induction of the amyloid cascade, and expression of inflammatory mediators [5]. Notably, the amyloid hypothesis states that amyloid β (Aβ) plays a critical role in AD pathogenesis and triggers several lesion events (including tau pathology, inflammation, synaptic dysfunction, and neuronal degeneration), eventually leading to the development of AD [6]. Aβ aggregates cause neurotoxicity and induce neuronal apoptosis by activating the caspase signaling pathway and promoting mitochondrial dysfunction [7]. Early intracellular accumulation of Aβ is observed in AD-susceptible brain regions, which precede the formation of NFT and senile plaques [8]. Furthermore, intracellular Aβ deposition is critical for the development of neuronal apoptosis and synaptic impairment [9].

Sesame (*Sesamum indicum* L.), a traditional crop cultivated worldwide, was used more than 4000 years ago as a highly valuable oil crop in Babylon, India, and Assyria, with different traditional medicinal applications [10]. Sesame seeds are a source of very high-quality oil, which contains several polyphenols that are characterized by their resistance to oxidation; therefore, they are highly stable during storage and processing [11]. Numerous studies have documented the unique nature of sesame oil owing to the presence of high levels of bioactive antioxidant lignans, such as sesamin, sesamolin, sesaminol, and sesamolinol [12]. These compounds are responsible for antioxidant and anticancer activities [13], cardioprotection [14], neuroprotection [15,16], anti-inflammatory [17], and chemo-preventive and anti-aging properties [18]. In particular, sesamin and sesamolin possess neuroprotective effects against hypoxia or brain damage [19], and are powerful antioxidants that inhibit ultraviolet- and Fe^3+^/ascorbate-induced lipid peroxidation in the rat brain [20]. These studies suggest that the consumption of sesame seed and oil has health benefits, including neuroprotection. Enrichment of lignans in seeds potentiates the characteristics of sesame to improve human health. Plant breeding (including sesame breeding) has contributed to increased food availability through the development of new functional varieties by increasing the marker compound (e.g., sesame lignan), which indicates health benefits. Therefore, we studied the development of cultivars and breeding lines with various lignan levels. Among them, ‘Milyang 74 (KACC 88003BP)’ was artificially bred using ‘Gyeongbuk 22’, which contains a high lignan content and short stem length, and ‘YCS71’, which has a high lignan content and fast maturity as parental lines [21].

**Figure 1:**
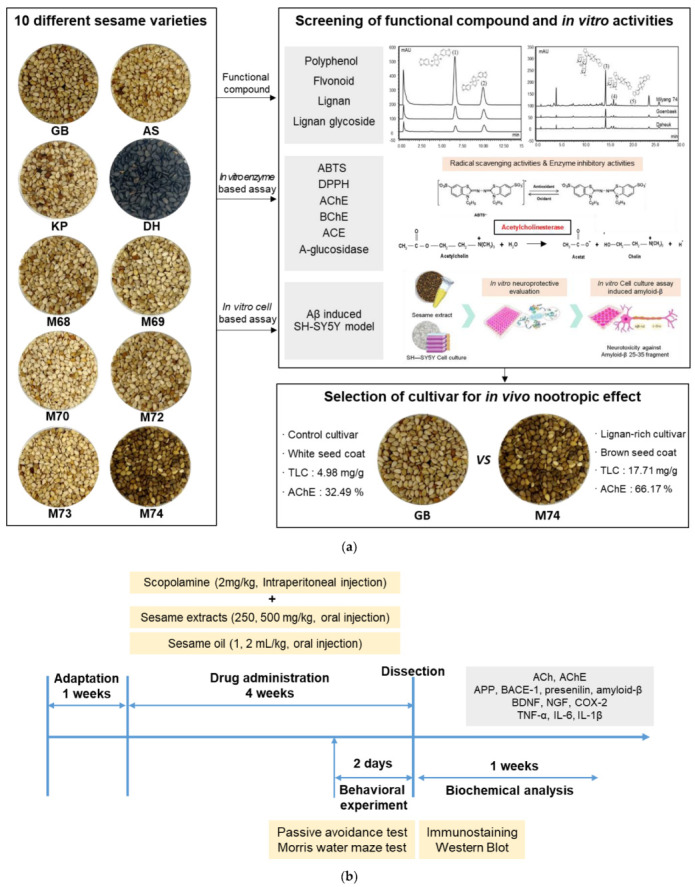
Schematic diagram. (**a**) Screening of functional compound and in vitro bioactive activities in different sesame varieties, (**b**) animal experiment design.

Although varieties and lines containing varying lignan contents have been developed, studies on the differences in cognitive function improvement between sesame extract and oil due to lignan content variation are insufficient. The objective of this study was to (a) investigate acetylcholinesterase inhibitory activities in vitro in 10 kinds of sesame varieties according to their lignan content and determine the neuroprotective effects of lignan-rich sesame varieties in amyloid-β-induced SH-SY5Y cells, and (b) to evaluate the in vivo nootropic effects of sesame extracts and oil from lignan-rich varieties in scopolamine (2 mg/kg)-induced memory impaired mice (Figure 1). Our study suggests that these extracts may be developed as functional foods to delay the onset of cognitive impairment, including AD, in middle-aged and older-adults.

## 2. Materials and Methods

### 2.1. Material and Chemicals

This study included four sesame cultivars: *Sesamum indicum* L. cv. Goenback, cv. Ansan, cv. Koppom, and cv. Daheuk. Additionally, six types of sesame lines containing various lignan contents (Milyang 68, Milyang 69, Milyang 70, Milyang 72, Milyang 73, and Milyang 74) were grown at the National Institute of Crop Science in Miryang, South Korea, during the 2020 growing season.

### 2.2. Preparation of Crude Extracts and Sesame Oil

Powdered samples (4 g) were extracted twice with 80% methanol (80 mL) at room temperature (25 °C) for 24 h utilizing an agitator. The extracts were subsequently filtered and concentrated using a rotary evaporator under vacuum, freeze-dried, and kept at −20 °C in an ultralow temperature freezer. Sesame seeds for each variety were washed and the moisture content was adjusted to 3–4% in a dryer at 30 °C. Sesame seeds (1 kg) were roasted for 20 min at 180 °C for each variety using an automatic frying pot (Dongbang Machinery Co., Daegu, Korea), cooled at room temperature for 30 min, and stored at 4 °C. Sesame oil was extracted from roasted sesame seeds using a hydraulic oil press machine (Dongbang Machinery Co., Daegu, Korea) under the same conditions. The internal temperature was set to 60 °C, while the maximum pressure for mechanical hydraulic press extraction was 60 MPa and held at that pressure for 20 min. The obtained oils were stored overnight at −4 °C until two distinct layers formed: a clear top oil layer and a viscous gummy bottom layer. The clear top layer was collected and stored at −20 °C in an ultralow temperature freezer.

### 2.3. Functional Compound Analysis of Different Sesame Varieties

Total polyphenol and flavonoid levels were measured as described previously [22]. The finding was represented as mg of gallic acid and catechin equivalents per g of sesame (mg GAE/g sesame, mg CE/g sesame). The lignan and lignan glycoside composition (sesamin, sesamolin, sesaminol, sesaminol-diglucoside, and sesaminol-triglucoside) of each cultivar was determined using high-performance liquid chromatography (HPLC) [23].

### 2.4. Antioxidant and Cholinesterase Inhibitory Activities of Different Sesame Varieties In Vitro

The radical scavenging activities of 2,2-azinobis (3-ethyl benzothiazoline)-6-sulfonic acid (ABTS) and 1,1-diphenyl-2-picrylhydrazyl (DPPH) were determined according to Woo et al. [22]. Trolox-equivalent antioxidant capacity (TEAC) in mg TE/g sesame (ER) was used to express the radical scavenging activities. The enzymatic inhibition of acetylcholinesterase (AChE) and butylcholinesterase (BChE) was evaluated in a microplate using a spectrophotometric method with an acetylcholinesterase inhibitor screening kit (Sigma, MA, USA) [24]

### 2.5. Neuroprotective Effect in Amyloid-β Induced SH-SY5Y Cells of Lignan-Rich Sesame Cultivars

Human neuroblastoma SH-SY5Y cells were obtained from the Korean Cell Line Bank (Seoul, Korea). In an incubator with a 5% CO_2_ environment at 37 °C, SH-SY5Y cells were kept alive in Dulbecco’s modified Eagle’s medium (DMEM) supplemented with 10% fetal bovine serum, 100 U/mL penicillin, and 50 g/mL streptomycin. Cells were subcultured with 0.05% trypsin-EDTA and phosphate-buffered saline (PBS). The toxicity of amyloid β-protein fragment 25–35 (Aβ_25-35_; 6.25, 12.5, 25, 50, and 100 μM) and sesame extracts (25, 50, 100, and 200 μg/mL) of different varieties (cv. Goenback, cv. Daheuk, cv. Milyang 74) was analyzed in human neuroblastoma SH-SY5Y using the MTT (3-[4,5-dimethylthiazol-2-yl]-2,5 diphenyl tetrazolium bromide) assay [25]. SH-SY5Y cells were seeded in a 96 well plate at a density of 2 × 10^4^ cells/well to determine the cytoprotective effects against Aβ_25-35_ aggregate (25 μM). After 24 h, each sesame extract concentration (50, 100, 200, and 400 μg/mL) was administered to the cells for 2 h, and Aβ_25-35_ aggregate (25 μM) was added. After incubation for 24 h, cell viability was determined using the MTT assay as previously described. Intracellular reactive oxygen species (ROS) levels were quantified using a DCFH-DA fluorescent probe as previously described [26]. Fluorescence intensity corresponding to intracellular ROS generation was measured with a fluorescence spectrophotometer for 2 h at an excitation wavelength of 485 nm and an emission wavelength of 530 nm. Cells were harvested and lysed using radioimmunoprecipitation assay buffer (RIPA, Sigma, MA, USA) to determine lipid peroxidation and antioxidant enzyme activity. The lysates were centrifuged at 10,000× *g* for 10 min at 4 °C, and the supernatants were utilized for protein and lipid peroxidation tests as well as antioxidant enzyme assays [27].

### 2.6. Treatment of Scopolamine-Induced ICR Mice with Extracts and Oil from Lignan-Rich Sesame Cultivars

Male ICR mice (6-weeks old; 29–30 g) were purchased from Hanabio, Inc. (Gyeonggi-do, Korea) and housed in a regulated environment (20–23 °C, 40–70% relative humidity, 12 h light/dark cycle, light period starting at 7:00 AM) with free access to food and water. Solid feed was provided to the experimental animals. Feed intake, drinking water, and body weight were measured twice a week. This study was approved by the Institutional Animal Care and Use Committee (No. SEMI-21-005, IACUC) of the Southeast Medi-Chem Institute (SEMI). Mice were randomly divided into 11 groups (*n* = 7): 1, normal (0.9% saline); 2, control (2 mg/kg scopolamine, intraperitoneal injection); 3, positive control (2 mg/kg scopolamine + 0.75 mg/kg donepezil, oral administration); 4, GBE-250 (2 mg/kg scopolamine + 250 mg/kg Goenback extract, oral administration); 5, GBE-500 (2 mg/kg scopolamine + 500 mg/kg Goenback extract, oral administration); 6, GBO-1 (2 mg/kg scopolamine + 1 mL/kg Goenback oil, oral administration); 7, GBO-2 (2 mg/kg scopolamine + 2 mL/kg Goenback oil, oral administration); 8, M74-250 (2 mg/kg scopolamine + 250 mg/kg M74 extract, oral administration); 9, M74-500 (2 mg/kg scopolamine + 500 mg/kg M74 extract, oral administration); 10, M74O-1 (scopolamine 2 mg/kg + M74 oil 1 mL/kg, oral administration); and 11, M74-2 (scopolamine 2 mg/kg + M74 oil 2 mL/kg, oral administration). The details of In vivo experimental design and results of body weight (g), tissue weight (g), and serum biochemistry analysis for experiment was presented in Supplementary Tables S3 and S4. Scopolamine (2 mg/kg, Sigma-Aldrich Co., St. Louis, MO, USA) was dissolved in saline and administered 30 min before oral injection of sesame extract and oil. All extracts and oils used for treatment were diluted in saline and orally injected for 4 weeks before the passive avoidance and water maze tests.

### 2.7. Memory Evaluation Using a Passive Avoidance Test and Water Maze Test Using ICR Mice

The passive avoidance task of scopolamine-induced memory impaired mice was measured using a shuttle box apparatus (GEMINI Avoidance system, San Diego Instruments, San Diego, CA, USA) to confirm memory loss and AD induction and to evaluate long-term memory improvement effects of extract and oil from the lignan-rich sesame cultivar, ‘Milyang 74’. The passive avoidance box comprised a light and dark chamber (25 × 15 cm), and a connecting guillotine door (10 × 10 cm) was located between the rooms. Mice were placed in the light chamber for 1 min with the door closed for adaption; the door was then opened, and the mice were returned to the dark chamber due to their preference for gloomy locations. When the mice entered the dark compartment, the door was closed, and an electric foot-shock (0.5 mA, 3 s) was delivered through the stainless-steel rods with a constant current shock generator. The latency and frequency of mice entering the dark compartment and the duration of stay in the dark chamber for 60 s was measured after 24 h. The entire experiment was performed in a separate room equipped with ventilation and by the same experimenters, taking into account the time interval and cleaning of the box between tests.

The Morris water maze test was performed to evaluate the spatial memory improvement effects of the extract and oil from the lignan-rich sesame cultivar (‘Milyang 74’) on scopolamine-induced memory impairment in mice. The Morris water maze used in this study was a round black cylinder (round tank) of 100 cm diameter and 30 cm depth, with an escape platform placed on a metal basis. The tank was filled with tap water (20 ± 2 °C) so that the escape platform was submersed in the water (1 cm below the surface). Mice were trained eight times in four consecutive days to become acquainted with the Morris water maze test. After the mice were left on the platform for 10 s to memorize their position, the experiment was repeated for 120 s. Escape latency time was evaluated for spatial memory improvement by recording the time spent on the platform, and measuring the swimming patterns.

### 2.8. Immunohistochemistry of AChE, ACh, TNF-α, IL-6, and IL-1β Levels in Mouse Brain Tissue Extracts

Mice were anesthetized with CO_2_ gas and immediately decapitated following the behavioral tests, and exsanguinated from the abdominal aorta after laparotomy. The liver, spleen, kidney, and brain tissues were harvested and weighed, and the whole brain was dissected on ice for immunostaining and further biochemical analysis. Immunostaining was used to detect neuronal changes in brain tissue fixed with 4% paraformaldehyde, rinsed, dehydrated, paraffin-embedded, and sliced into sections with a thickness of 4 μm. An optical microscope (Olympus, Tokyo, Japan) was used to obtain six microscopic images from three sections of each immuno-stained group at 400× magnification. For biochemical analysis, some brain tissue was homogenized with 12.5 mM sodium phosphate buffer (400 mM NaCl, pH 7.0) and centrifuged at 14,000 rpm for 10 min at 4 °C. Major biomarkers related to cholinergic function in mouse brain tissue (such as acetylcholinesterase or AChE, MAK119, Sigma-Aldrich, USA; and acetylcholine or Ach, MBS733116, Mybiosource, San Diego, CA, USA) were determined using an enzyme linked immunoassay (ELISA) kit. In addition, the major secreted cytokines related to inflammation in mouse brain tissue extracts (tumor necrosis factor α or TNF-α, interleukin-6 or IL-6, and interleukin-1β or IL-1β) were determined via ELISA. The TNF-α, IL-6, and IL-1β ELISA kits were used (R&D Systems), according to the manufacturer’s instructions.

### 2.9. Western Blot Analysis of Protein Expression Related to Amyloid-β Accumulation, Neuronal Regeneration, and Inflammation in Mouse Brain Tissue Extracts

Protein expression related to amyloid-β accumulation (APP, BACE-1, presenilin, amyloid-β), neuronal regeneration (NGF, BDNF), and inflammation (COX-2) was investigated using Western blot analysis. A bicinchoninic acid (BCA) protein assay kit (Thermo Fisher Scientific, Waltham, MA, USA) was used to assess protein concentration, followed by Western blot analysis. Protein (15 μg) extracted from brain tissue was separated using 10% sodium dodecyl sulfate (SDS-PAGE) and transferred to a polyvinylidene difluoride (PVDF) membrane. The membrane was blocked in a buffer containing antibodies of APP (amyloid precursor protein, 1:1000, 2452 S, Cell Signaling, Danvers, MA, USA), BACE-1 (β-secretase 1, 1:1000, 5606 S, Cell Signaling, USA), presenilin (1:1000, 5643 S, Cell Signaling, USA), amyloid-β (1:1000, MOAB-2, NOVUS Biological, USA), NGF (nerve growth factor, 1:1000, PA5-29425, Invitrogen, Waltham, MA, USA), BDNF (brain-derived neurotrophic factor; 1:1000, 47,808 S, Cell Signaling, USA), and COX-2 (cycloxygenase-2, 1:1000, 12,282 S, Cell Signaling, USA) at 4 °C overnight. The membrane was incubated with specific secondary antibodies (1:2000) at room temperature for 1 h, then further incubated with enhanced chemiluminescence (ECL) substrate and the bands were detected using a Chemi-Doc XRS system. Protein loading was evaluated using anti-β-actin antibody.

## 3. Results and Discussion

### 3.1. Screening of Functional Compounds in Different Sesame Varieties

**Figure 2:**
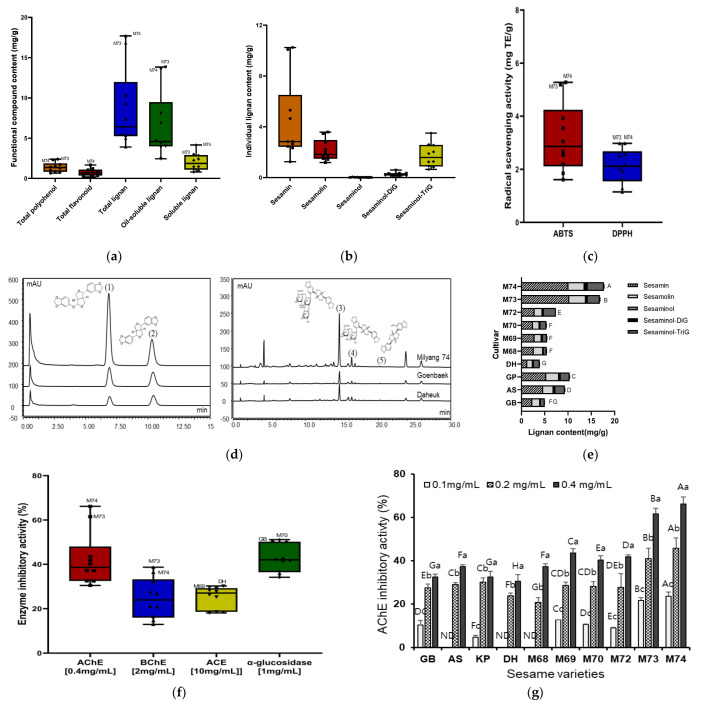
Functional compound contents, antioxidant activity, and enzyme inhibitory activity from 10 sesame varieties. Values are the mean ± SD of 3 replicates. Different capital letters and small letters in the same items indicate a significant difference (*p* < 0.05) among different varieties and extraction concentration, respectively. (**a**) Functional compound contents: total polyphenol content, total flavonoid content, total lignan content, oil−soluble lignan, soluble lignan (**b**); (**e**) individual sesame lignan composition: sesamin, sesamolin, sesaminol, sesaminol-diglucoside, and sesaminol−triglucoside. (**c**) Antioxidant activities: 2,2′−azino−bis3−ethylbenzothiazoline−6−sulphonic acid (ABTS) radical scavenging activity, 2,2-diphenyl−1−picrylhydrazyl (DPPH) radical scavenging activity. (**d**) High performance liquid chromatography (HPLC) chromatogram of sesame lignan: (1) sesamin, (2) sesamolin, (3) sesaminol-−triglucoside, (4) sesaminol−diglucoside, (5) sesaminol. (**f**) Enzyme inhibitory activities: acetylcholinesterase (AChE), butylcholinesterase (BChE), angiotensin converting enzyme (ACE), and α-glucosidase (AG) inhibitory activity. (**g**) Acetylcholinesterase (AChE) inhibitory activity depending on 10 varieties and extracts concentration (0.1, 0.2, 0.4 mg/g): Goenbaek (GB), Ansan (AS), Koppom (KP), Daheuk (DH), and Milyang 68, 69, 70, 73, 74.

Lignan and phenolic compounds are major antioxidants found in fruits, vegetables, and grains. Therefore, we measured polyphenol, flavonoid, lignan, and lignan glycoside levels and their bioactive contributions in 10 sesame varieties before evaluating the nootropic effects of lignan-rich sesame cultivars. The distribution of total polyphenol content (TPC), total flavonoid content (TFC), lignan, and lignan glycoside content was determined in 4 kinds of sesame cultivars (cv. Goenback (GB), cv. Ansan (AS), cv. Koppom (KP), and cv. Daheuk (DH)), and six sesame lines (Milyang 68 (M68), Milyang 69 (M69), Milyang 70 (M70), Milyang 72 (M72), Milyang 73 (M73), and Milyang 74 (M74)) (Figure 2a,b and Supplementary Table S1). The variation in lignan (sesamin and sesamolin) and lignan glycoside (sesaminol, sesaminol-diglycoside, sesaminol-triglycoside) content between the 10 sesame varieties was greater than the total polyphenol and flavonoid contents. In particular, sesamin and sesaminol-triglucoside contents were varyingly distributed in the range of 1.25–10.25 mg/g and 0.82–3.50 mg/g, respectively. The total polyphenol and flavonoid contents of different sesame varieties were 0.66–2.39 mg GAE/g and 0.36–1.22 mg CE/g, respectively. The lines of M73 and M68 had the greatest total polyphenol content (2.39 mg GAE/g) as well as the highest total flavonoid content (1.22 mg CE/g). These contents were generally higher in the new breeding lines than those in the conventional sesame cultivars. The lignan and lignan glycoside profiles of sesame varieties were analyzed using HPLC (Figure 2e). This revealed the presence of two lignans and three lignan glycosides (sesamin, sesamolin, sesaminol, sesaminol-diglucoside, and sesaminol-triglucoside) among the six lignans comprising the standard (STD; 280 nm). The total lignan content (TLC) was 3.90–17.71 mg/g, and the highest TLC (17.71 mg/g) was observed in M74 extracts. The total oil-soluble lignan content, (including sesamin and sesamolin) was 4.02–10.08 mg/g. The total soluble lignan content (including sesaminol, sesaminol-diglucoside, and sesaminol-triglucoside) was 0.96–4.15 mg/g. The highest total oil soluble lignan amount was detected in M73 (13.83 mg/g), followed by M74 (13.56 mg/g), KP (8.09 mg/g), and AS (6.89 mg/g). The highest total soluble lignan amount was detected in M74 (4.15 mg/g), followed by M73 (3.00 mg/g), M72 (2.86 mg/g), and AS (2.41 mg/g). According to Shi et al. [28], the TLC in 100 sesame seeds and 56 sesame oil samples in China ranged from 2.52 to 12.76 and 3.38 to 11.53 mg/g. Therefore, M73 or M74 sesame lines have significantly higher lignan content than general sesame cultivars. Sesamin and sesamolin have neuroprotective effects against hypoxia and brain damage [19]. Sesamol is a powerful antioxidant that inhibits ultraviolet and Fe^3+^/ascorbate-induced lipid peroxidation in rat brain [20]. It also possesses neuroprotective, hepatoprotective, anti-inflammatory, chemopreventive, and anti-aging properties [29]. These variations in phenolics and individual lignan levels in different varieties of sesame in this study are thought to affect physiological characteristics, including in vitro anti-cholinesterase activity, anti-neurotoxicity in amyloid-β induced SH-SY5Y cells, in vivo nootropic effects in scopolamine-induced memory impaired mice, and contain antioxidant characteristics.

### 3.2. Screening of Antioxidant Activity and Enzyme Inhibitory Activity in Different Sesame Cultivars

Sesame lignans, such as sesamin, sesamolin, and sesaminol are known to have significant antioxidant activity and thus play an important part in the prevention of the development of many chronic diseases [12]. In the present study, the extracts from 10 different sesame varieties were evaluated for their antioxidant capacity using ABTS and DPPH radical in vitro assay (Figure 2e, Supplementary Table S2). ABTS and DPPH radicals are used widely to evaluate the free radical-scavenging activities of hydrogen-donating and chain-breaking antioxidants in many plant extracts [30].

ABTS and DPPH radical scavenging activities of extracts from 10 different sesame varieties were distributed in the ranges of 1.61–5.28 and 1.15–2.98 mg TE/g, respectively. The ABTS and DPPH were highest in M74 (5.28 and 2.98 mg TE/g, respectively), followed by M73, M68, and GB, and were generally higher in the new breeding lines which contained high concentrations of total phenols, flavonoids, individual lignan, and lignan glycoside than that in the conventional sesame cultivars. Many similar reports on antioxidant activities were found based on the distribution of functional compounds including lignan in various sesame varieties. Othman et al. [31] reported that extracts from white sesame seeds had a greater antioxidant capacity than extracts from gold sesame seeds and attributed this to a difference in bioactive compound concentration. However, in the present study, M74 with brown sesame seed coat has the highest antioxidant capacity; it is thought that the excellent antioxidant activity is due to functional ingredients such as lignan and lignan glycosides regardless of seed coat color. Furthermore, Ide et al. [32] compared the physiological activities of sesame seeds with different concentrations of lignans in rats and found that sesame seeds rich in lignans, irrespective of composition of lignans, greatly affect hepatic fatty acid oxidation and serum triacylglycerol levels. Although antioxidant activities of sesame varieties containing varying lignan contents have been reported in many investigations, in vitro and in vivo studies on the difference in cognitive function improvement between sesame extract and oil due to lignan content variation are insufficient.

Enzyme inhibitors are essential for the treatment of a wide range of diseases, including diabetes mellitus, hyperpigmentation, and illnesses linked to the nervous system, such as stroke, Alzheimer’s disease, vascular dementia, and Parkinson’s disease [33]. In this study, the enzyme inhibitory properties of extracts from 10 different sesame varieties were assessed against AChE, BChE, ACE, and α-glucosidase (Figure 2f, Supplementary Table S2). In total, the enzyme inhibition capacity related to cholinesterase (AChE, BChE) of extracts from 10 different sesame varieties is more effective in low concentration than ACE and α-glucosidase associated with hypertension and diabetes. Especially, acetylcholinesterase (AChE) inhibition activities of extracts from 10 different sesame varieties have remarkable capacity with a range of 32.49–66.17% at low concentration (0.4 mg/mL). This inhibition ability was enhanced with increasing polyphenol, flavonoid, individual lignan, and their glycoside. Accordingly, AChE inhibitory activity was highest in M74 (66.17%), followed by M73 (61.49%), M69 (43.57%), and M72 (41.78%), and were generally higher in the new breeding line as seen in the results of antioxidant capacity. In general, acetylcholinesterase modulates acetylcholine concentration in cholinergic synapses, which is an important cognitive process [34]. However, memory deficiency is affected by various mechanisms with toxic effects including the formation of amyloid beta plaques, cholinergic deficits, increased acetylcholinesterase enzyme levels, oxidative stress, and neuroinflammation [35]. Therefore, based on these screening results regarding antioxidant and AChE inhibition capacity associated with cognitive functions, anti-neurotoxicity in amyloid-β induced SH-SY5Y cells, and in vivo nootropic effect produced in scopolamine-induced memory impaired mice, was investigated using lignan-rich ‘Milyang 74’ sesame cultivar among various varieties.

### 3.3. Neuroprotective Effect of Lignan-Rich Sesame Cultivar in Amyloid-β Induced SH-SY5Y Cell

**Figure 3:**
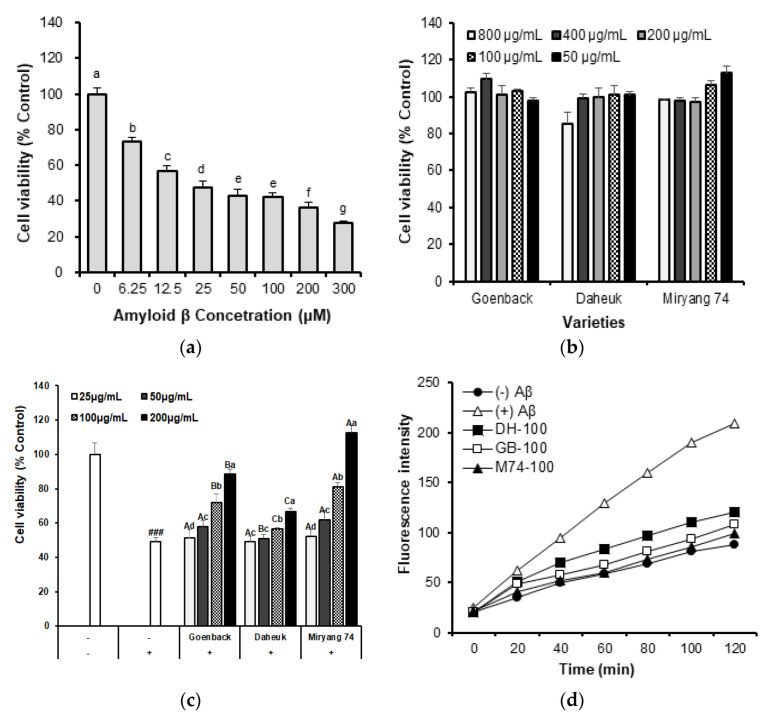
Effect of lignan−rich ‘Milyang 74’ sesame cultivar on neuroprotective activities (25, 50, 100, 200 μg/mL) in amyloid−β−induced SH-SY5Y cell death compared to ‘Goenback’ and ‘Daheuk’ control cultivars. Values are means ± SD of 3 replicates. ^###^ *p* < 0.001 significant difference compared to control. Different capital letters and small letters in the same items indicate a significant difference (*p* < 0.05) among different varieties and extraction concentration, respectively. (**a**) Cell viability, (**b**) neuroprotective effect, (**c**) intracellular reactive oxygen species (ROS) levels, (**d**) fluorescence intensity for ROS in amyloid−β−induced SH−SY5Y cells. Different capital letters and small letters in the same items indicate a significant difference (*p* < 0.05) among different cultivars and sample treatment concentrations, respectively.

The neuroprotective effects of lignan-rich ‘M74’ sesame cultivar extracts on amyloid- *β(Aβ)* induced SH-SY5Y cells was investigate to evaluate cell viability, reduction of reactive oxygen species (ROS) inhibition, malondialdehyde (MDA) contents, and activity of antioxidant enzyme (glutathione reductase, GR; glutathione peroxidase, GPx; SOD; catalase, CAT). The SH-SY5Y cell line has been used widely in experimental neurological studies, including analysis of neuronal differentiation, metabolism, and function related to neurodegenerative processes, neurotoxicity, and neuroprotection [36]. For anti-neurotoxicity study of sesame extracts, preliminary experiments were first performed to determine the toxic concentrations of sesame extract Aβ_25-35_ fragment on SHSY5Y cells by 3-[4,5-dimethylthiazol-2-yl]-2,5-diphenyl-tetrazolium bromide (MTT) assay (Figure 3a,b). The exposure of SH-SY5Y cells to Aβ_25-35_ resulted in an induction of 50% of cytotoxicity compared with the control cells. Therefore, the 25 μM Aβ_25-35_ fragment at 24 h incubation was chosen to induce cell death for subsequent experiments. The cell viability of SH-SY5Y neuroblastoma was 49.01% after exposure to the 25 μM Aβ_25-35_ fragment for 24 h. Then, we analyzed the cytoprotective effects of sesame extracts of the three different cultivars including ‘M74’ against Aβ-caused toxic damage (Figure 3c). These findings show that treatment with the 25 μM Aβ_25-35_ fragment reduced cell viability by 49.01% as compared to control cells. However, independent of cultivar identification, pretreatment with sesame extract at 50–400 g/mL dramatically enhanced cell viability in a dose-dependent manner. The strongest protective effects of the A-induced SH-SY5Y cell were reported in sesame extracts from the lignan-rich ‘M74’ cultivar; at concentrations of 100 and 200 g/mL, these extracts enhanced cell viability to 81.23% and 112.74%, respectively. Moreover, treatment with Aβ significantly increased oxidative cellular stress and damage, increasing ROS production in SH-SY5Y cells by 209.28% compared to that in the untreated control cells.

However, pretreatment with sesame extracts at concentrations ranging from 50 to 400 g/mL dramatically reduced intracellular ROS levels in Aβ-induced SH-SY5Y cells in a dose-dependent manner. M74 extracts reduced the ROS levels in Aβ-induced cells from 209.28% to 98.98% at a concentration of 100 g/mL (Figure 3d). We also measured intracellular MDA concentrations, a lipid peroxidation indicator, in the Aβ-induced SH-SY5Y cells (Table 1). Intracellular antioxidant enzymes perform crucial functions in the oxidative damage defense process. In SH-SY5Y cells, oxidative stress induced by treatment with Aβ A increased MDA concentrations and the enzyme activities of GR, GPX, CAT, and SOD (Table 1). However, these increases were reversed by pretreatment with sesame extracts at 100 μg/mL. Among them, the sesame extracts of the lignan-rich ‘M74’ cultivar were the most effective in reversing the increase in the MDA concentration and activities of GR, GPx, CAT, and SOD. A recent study suggested that amyloid-β is the main neurotoxic factor that induced neuronal death and oxidative stress in the early stages of AD [37]. The present study investigated whether extracts from the lignan-rich ‘M74’ cultivar improve cell viability in the presence of Aβ, and reduce ROS levels from oxidative stress, and suppressed lipid peroxidation and intracellular antioxidant enzymes. According to a previous study, Ben Othman et al. [38] reported that the water-soluble fractions from sesame seeds can protect neuroblast cells against both extracellular and intracellular oxidative stress. Furthermore, Xu et al. [39] reported that Aβ-induced injury to SH-SY5Y cells resulted in the production of intracellular ROS and cellular death and sesamin attenuated disadvantages after oxidative stress. Various studies have reported the neuroprotective effect of sesame lignan such as sesamin, sesamolin, and sesaminol in Aβ-induced SH-SY5Y model, mainly due to their antioxidant ability. However, no research has been conducted to determine the effect of sesame extracts from lignan-rich cultivars on neuroprotective properties against oxidative stress in A-induced SH-SY5Y mutant mice. We hypothesized that the ‘M74’, which contains high amounts of individual lignan and lignan glycosides, can provide strong protection against oxidative damage in SH-SY5Y cells by modulating ROS production, MDA generation, and antioxidant enzyme activities.

### 3.4. Effect of Lignan-Rich Sesame Cultivar Pretreatments on the Improvements of Impaired Memory Functions in Scopolamine-Treated ICR Mice

**Figure 4:**
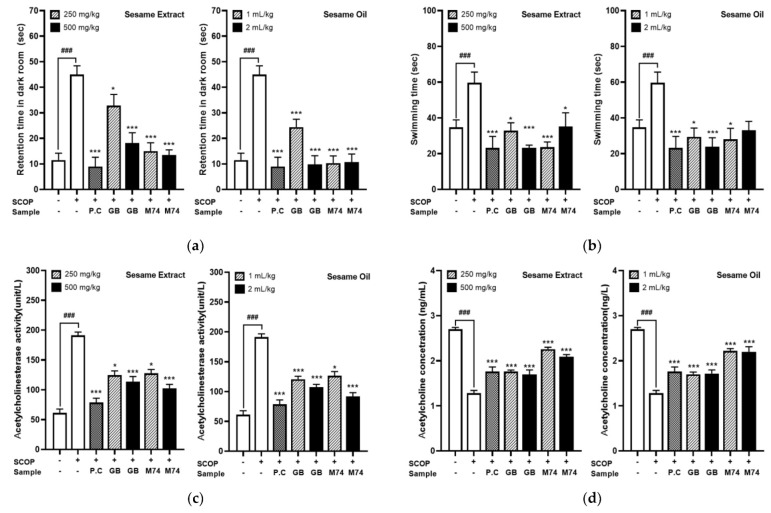
Effect of lignan−rich sesame cultivar (‘Milyang 74’) and control cultivar (‘Goenback’) pretreatments (sesame extract: 250, 500 mg/kg; sesame oil: 1, 2 mL/kg) on the improvements of impaired memory functions, AChE activity, and ACh concentration in SCOP (scopolamine) −treated ICR mice. Values are means ± SD of 7 replicates. ^###^ *p* or *** *p* < 0.0001, * *p* < 0.05 significant difference compared to SCOP−treated control. (**a**) Passive avoidance test, (**b**) water maze test, (**c**) acetylcholinesterase activity, (**d**) acetylcholine concentration.

Behavioral alterations were examined using passive avoidance test and Morris water maze test to evaluate the influence of sesame extracts and oil from the lignan-rich Milyang 74 cultivar on memory function improvements in scopolamine-treated ICR mice. The scopolamine-treated group presented a significant increase in retention time for escaping the electric foot shock in the dark chamber compared to the control group (11.5 s vs. 45.0 s) in the passive avoidance test (*p* < 0.0001, Figure 4a). Meanwhile, a remarkable reduction in the escape latency was observed in the positive control (donepezil, 0.75 mg/kg, 9.0 s), sesame extracts (250 or 500 mg/kg, 13.5–32.8 s), and sesame oil (1 or 2 mL/kg, 9.8–24.4 s) administered group (*p* < 0.0001). The avoidance time was shortened to a similar level to that of the positive control group (donepezil, 0.75 mg/kg, 9.0 s), even when sesame extract (250 mg/kg, 15.0 s) and oil (1 mL, 10.3 s) from Milyang 74 were administered at low concentrations. These results showed decreases in time of 2.2 and 2.4 times compared to the sesame extracts (250 mg/kg, 32.8 s) and oil (1 mL, 24.4 s) from the ‘Goenback’ general sesame cultivar, and it was confirmed that intake of sesame extracts and oil from ‘Milyang 74’ improves long-term memory functions in scopolamine-treated ICR mice. The scopolamine-treated group demonstrated a marked increase in escape latency compared to the vehicle control group (34.8 s vs. 60.0 s, *p* < 0.001) in the Morris water maze test (Figure 4b).

A remarkable reduction in escape latency was observed in the positive control (donepezil, 0.75 mg/kg, 23.3 s), sesame extracts (250 or 500 mg/kg, 23.2–35.2 s), and sesame oil (1 or 2 mL/kg, 23.8–33.0 s) administered groups. Regarding lignan content of the sesame cultivar, the difference in spatial perception in the Morris water maze test was not as evident as that in the long-term memory ability in the passive avoidance test; however, the escape latency time decreased in all treatment groups regardless of cultivar and injection concentration. Mohamed et al. [40] reported that sesame oil significantly improved learning and memory impairments induced by AlCl_3_. In addition, co-administration of sesame oil to AlCl_3_-intoxicated rats significantly reduced AChE activity and improved cognition, memory performance, and locomotor activity. Sesame oil also improves learning and memory [41,42] in diabetic rats [43] and scopolamine-treated rats [44]. Various studies have attempted to improve the cognitive function of sesame oil; however, there are no studies on behavioral alterations according to the variation in lignan concentration of different sesame cultivars. These behavioral alterations of AD are associated with memory deficiency with toxic events, including the formation of amyloid beta plaques, cholinergic deficits, increased acetylcholinesterase enzyme levels, oxidative stress, and neuro-inflammation [35]. In this study, cholinergic deficit characteristics and protein expression related to amyloid-β accumulation, neuronal regeneration, and neuro-inflammation were analyzed to investigate the reason for the improvement in behavioral alterations in a scopolamine-induced ICR mouse model according to the lignan content of sesame cultivars.

### 3.5. Effect of Lignan-Rich Sesame Cultivar Pretreatments on Acetylcholine (ACh) Concentration and Acetylcholinesterase (AChE) Activity in Mouse Brain Tissue Extracts

Cholinergic deficiency induced by cholinergic neuronal degeneration greatly contributes to memory loss and cognitive impairment and is associated with the severity of AD [45]. Acetylcholine is regarded as a critical neurotransmitter involved in memory [46]. ChAT synthesizes ACh in cholinergic neurons, whereas AChE hydrolyzes ACh in synapses. The expression level of AChE and ChAT modulate ACh concentration in cholinergic synapses, which is important in the memory process [34]. The AChE inhibitors DNZ, donepezil, rivastigmine, and galantamine are approved as therapies for AD [47]. However, these drugs have various side effects (including insomnia) and research on safer alternatives is ongoing [48]. Therefore, ACh concentration and AChE activity was measured to assess the effects of sesame extracts and oil from the lignan-rich ‘Milyang 74’ cultivar on preventing AD by inhibiting the reduction in ACh levels caused by scopolamine in mouse brain regions, including the prefrontal cortex, hippocampus, and entorhinal cortex (Figure 4c,d). Scopolamine administration decreased ACh levels and enhanced AChE activity in mouse brain tissue extracts compared to that in controls and scopolamine can cause cholinergic system disorders and impairs learning and memory function in humans and rodents [49]. However, these alterations were attenuated by the positive control (donepezil), sesame extract, and oil pretreatment. In particular, this study found that scopolamine treatment decreased ACh levels and upregulated AChE activity in brain tissue compared with the controls. Furthermore, sesame extracts and oil from the lignan-rich cultivar (‘Milyang 74’) suppressed the decrease in ACh levels compared to the sesame extracts and oil from the general sesame cultivar, ‘Goenback’. Several studies have investigated the cognitive enhancement effects of sesame oil and sesame lignan on cholinergic regulation mechanisms. Mohamed et al. [40] reported that the co-administration of sesame oil to AlCl_3_-intoxicated rats dramatically lowered AChE activity, followed by improvements in cognition, memory function, and locomotor activity. Clinical research by Ito et al. shows that dietary supplements (including sesame lignans) improve cognitive function [50]. Sesamin improves cognition in diabetic rat models and lipopolysaccharide (LPS)-induced neuroinflammation through the amelioration of AChE levels [51]. Sesamol regulates dysfunction of the cholinergic system [52]. It normalizes the unbalanced cholinergic system by inhibiting AChE activity and improving ACh and ChAT activity. Recent research consistently shows that sesamol pretreatment balances cholinergic system disorder and increases the expression of M1 mAChR [53]. From these results, we hypothesized that lignan-rich cultivars with high amounts of sesamin, sesamolin, and sesamol have higher ACh levels and lower AChE activity in brain tissue and that general sesame cultivars lead to benefits in cognition and memory function.

### 3.6. Effect of Lignan-Rich Sesame Cultivar Pretreatments on Protein Expression Related to Amyloid-β Accumulation in Mouse Brain Tissue Extracts

**Figure 5:**
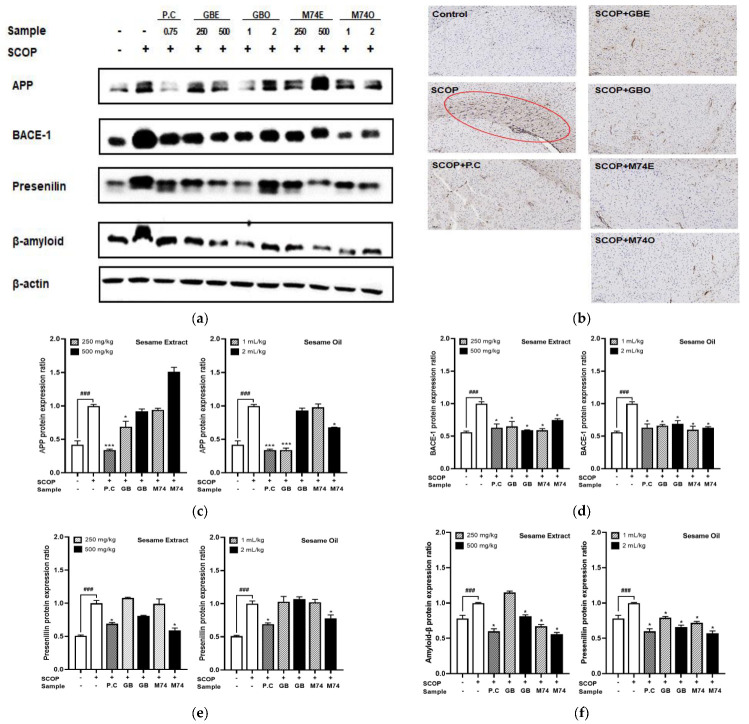
Effect of lignan-rich sesame cultivar (‘Milyang 74’) and control cultivar (‘Goenback’) pretreatments (sesame extract: 250, 500 mg/kg, sesame oil: 1, 2 mL/kg) on protein expression related to amyloid-β accumulation in mouse brain tissue extract of scopolamine-treated ICR mice. Values are means ± SD of 3 replicates. ^###^ *p* or *** *p* < 0.0001, * *p* < 0.05 significant difference compared to SCOP-treated control. (**a**) Western blot analysis of APP, BACE-1, presenilin, and amyloid-β, with GAPDH used as the loading control. (**b**) Glial fibrillary acidic protein (GFAP), (**c**) amyloid precursor protein (APP), (**d**) BACE-1 (**e**) presenilin, (**f**) amyloid-β.

Accumulation of amyloid-*β(*A*β*) causes apoptosis and oxidative stress, and consequently damages neurons and impairs cognitive function; it is one of the main pathogenic causes of AD [54]. The mechanisms of A*β* remain unclear; however, they are linked to aberrant enzyme cascades wherein A*β* generates neurotoxic species and contributes to AD progression [55]. A*β* is produced through the degradation of APP (amyloid-*β* precursor protein) by γ-secretase and BACE-1 [56]. The first step of APP cleavage by BACE generates C-terminal fragment (CTF) intermediates [57]. Subsequently, endopeptidase γ-enzyme cleaves CTF to produce A*β* and other metabolites [58]. Presenilins are a family of related multi-pass transmembrane proteins that constitute the catalytic subunits of the secretase intramembrane protease protein complex. Therefore, APP, BACE-1, and presenilin expression levels are expected to play an important role in A*β* production. We analyzed the expression of APP, BACE-1, presenilin, amyloid-β protein, and glial fibrillary acidic protein (GFAP) through immunostaining to investigate the influence of sesame extracts and oil from the lignan-rich cultivar (‘Milyang 74’) on the accumulation of A*β* caused by scopolamine in mouse brain regions. This indicates the severity of brain tissue damage. The normal control group showed few GFAP-positive cells in any hippocampal subregion, whereas scopolamine treatment resulted in a substantial number of GFAP-positive cells in all hippocampal subregions (Figure 5b); this signifies astrocyte activation. However, positive control (donepezil, 0.75 mg/kg), sesame extracts (250 or 500 mg/kg), and sesame oil (1 or 2 mL/kg) pretreatment attenuated scopolamine-induced astrocyte activation in all hippocampus subregions. There was a significant increase in the protein expression of APP (*p* < 0.0001), BACE-1 (*p* < 0.0001), presenilin (*p* < 0.0001), and A*β* (*p* < 0.0001) in the scopolamine-induced group compared to the controls (Figure 5c–f). Sesame extracts did not show a remarkable tendency in the expression of APP, BACE-1, presenilin, and Aβ regardless of the cultivar, whereas sesame oil derived from the lignan-rich cultivar (‘Milyang 74’) showed a concentration-dependent decrease in the expression of related Aβ accumulation. In other words, high doses of sesame oil (2 mL/kg) from the lignan-rich cultivar (‘Milyang 74’) suppressed the decrease in APP (*p* < 0.001), BACE-1 (*p* < 0.001), presenilin (*p* < 0.001), and Aβ (*p* < 0.001). A clinical study by Katayama et al. reported that sesamol and sesaminol improved learning and memory deficits induced by Aβ in the Morris water maze test [59]. Similar results showed that sesame oil downregulated amyloid-β accumulation in a rat model of AD [40]. Recovery from neuronal injury is a pathological pathway for various degenerative processes in AD [60]. Several factors, such as amyloid-β plaques, dysregulated calcium levels, glutamate, ischemia, inflammation, and oxidative stress, may trigger this process [61]. Therefore, we investigated various complex factors, including the regulation of cholinergic dysfunction, neuronal generation, inflammation, and protein expression related to amyloid-β accumulation.

### 3.7. Effect of Lignan-Rich Sesame Cultivar Pretreatments on Protein Expression Related to Neuronal Regeneration in Mouse Brain Tissue Extracts

**Figure 6:**
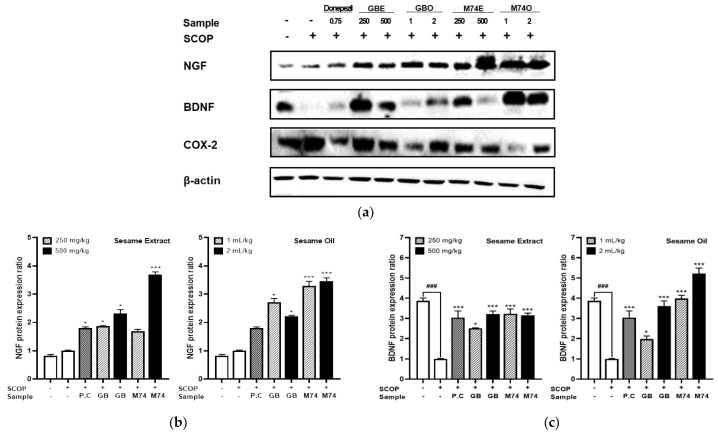

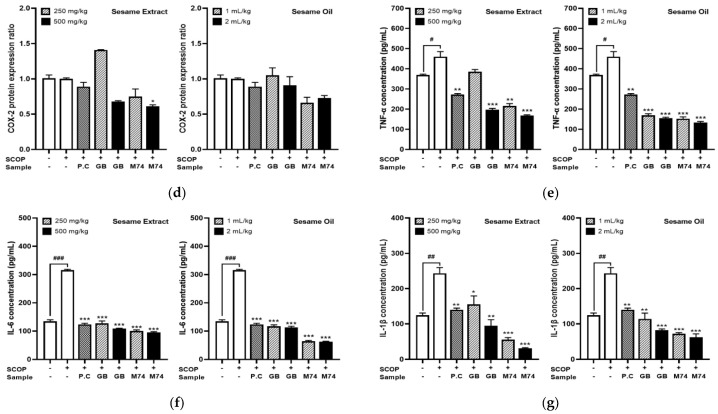
Effect of lignan−rich sesame cultivar (‘Milyang 74’) and control cultivar (‘Goenback’) pretreatments (sesame extract: 250, 500 mg/kg, sesame oil: 1, 2 mL/kg) on protein expression related to neuronal regeneration and inflammation. Values are means ± SD of 3 replicates. ^###^ *p* or *** *p* < 0.0001, ^##^ *p* or ** *p* < 0.001, ^#^ *p* or * *p* < 0.05 significant difference compared to SCOP-treated control. (**a**) Western blot analysis of NGF, BDNF, and COX-2 was performed with GAPDH used as the loading control. (**b**) NGF, (**c**) BDNF, (**d**) COX−2, (**e**) TNF−α, (**f**) IL−6, (**g**) IL−1β.

Growth or trophic factors, such as brain-derived neurotrophic factor (BDNF) and nerve growth factor (NGF), have neurogenic and neuroprotective effects [62]. Recent studies in animal models have reported that NGF deprivation causes AD-like pathologies, such as A*β* accumulation, tau hyper-phosphorylation, and synaptic dysfunction, and that NGF treatment can reverse these pathological changes [63]. BDNF is also essential for neurogenesis, neuroprotection, and synaptic plasticity [64]. Low BDNF mRNA and protein levels are observed in post-mortem AD brain tissues [65]. BDNF gene expression is regulated by extracellular signal-regulated protein kinase (ERK, a signaling molecule in the MAPK pathway) and cAMP response element binding protein (CREB, a transcription factor and downstream signaling molecule of ERK) [66]. CREB signaling is associated with memory augmentation, and disruption of CREB activity interrupts this process [67]. In this study, scopolamine administration decreased BDNF protein expression in mouse brain tissue compared to that in the controls. However, these alterations were attenuated by the positive control (donepezil), sesame extract, and oil pretreatment (Figure 6a–c). This study discovered that scopolamine treatment decreased NGF and BDNF expression in brain tissue compared to the control, whereas the administration of sesame extracts and oil from the lignan-rich cultivar (‘Milyang 74’) significantly increased NGF (sesame extracts and oil, *p* < 0.0001) and BDNF (sesame extracts, *p* < 0.05; sesame oil, *p* < 0.0001) expression by up to 2.3 times compared with sesame extracts and oil from the general sesame cultivar, ‘Goenback’. Recent studies show that the loss of BDNF or the ‘neurotrophic hypothesis of expression’ is an essential theory, and various lignans (including sesamin) upregulate neuronal regeneration factors. Sesamin improves BDNF and NT3 expression in the hippocampus of stress-induced mice [68], which might be partly explained by its beneficial effects on depressive-like behaviors. The interaction of sesamin treatment and stress factors had significant effects on the mRNA expression of BDNF and neurotrophin 3 (NT3). NT3, NT4, and NGF are a group of neurotrophic factors of the NGF family that exhibit activity on certain neurons of the peripheral and central nervous systems [69]. Therefore, we postulated that lignan-rich cultivars with high amounts of sesamin and sesamolin upregulate the expression of proteins related to neuronal regeneration (such as BDNF and NGF) in the brain tissue compared to general sesame cultivars, resulting in improved cognitive performance.

### 3.8. Effect of Lignan-Rich Sesame Cultivar Pretreatments on Protein Expression Related to Neuronal Inflammation in Mouse Brain Tissue Extracts

Neuronal inflammation is another pathogenic mechanism that contributes to memory loss caused by neurodegeneration. Pro-inflammatory cytokines and ROS generated by activated microglia and astrocytes at the site of inflammation may cause apoptosis and necrosis [70]. Furthermore, pro-inflammatory mediators generated by microglia and astrocytes stimulate each other to amplify the inflammatory signals in neurons. The main pro-inflammatory cytokines (TNF-α, IL-6, and IL-1β) are upregulated in AD brains, whereas the anti-inflammatory cytokines (IL-4, IL-10, and TGF-β) are downregulated in patients with AD [71]. A balance between pro- and anti-inflammatory mediators is critical for neuronal inflammation. Sesame lignan has a protective function against neuronal inflammation in different dementia models [72]. Therefore, we hypothesized that sesame extract and oil from the lignan-rich cultivar (‘Milyang 74’) might have neuroprotective effects against scopolamine-induced neuronal inflammation.

The modulatory influence of sesame extracts and oil from the lignan-rich cultivar (‘Milyang 74’) was investigated for the brain expression levels of TNF-α, IL-6, IL-1β, and Cox-2 to determine the effects on memory function recovery (Figure 6d–g). Scopolamine treatment significantly increased the concentrations of TNF-, IL-6, and IL-1 in mouse brain tissue extracts compared to the control (*p* < 0.0001). A large reduction was observed in the positive control (donepezil, 0.75 mg/kg), high dose sesame extracts (500 mg/kg), and sesame oil (2 mL/kg) administered group in the case of TNF-α and IL-6 (*p* < 0.0001). Moreover, IL-1β was slightly reduced in the positive control (0.75 mg/kg donepezil), high dose of sesame extract (500 mg/kg), and sesame oil (2 mL/kg) groups compared to the scopolamine-treated group. Particularly, even when sesame extracts (250 mg/kg) and oil (1 mL) from the lignan-rich ‘Milyang 74’ sesame cultivar were administered at low concentration, the IL-6 level was reduced to a similar level to that of the positive control group (donepezil, 0.75 mg/kg), similar to behavioral alterations such as passive avoidance test. An early study found increased levels of pro-inflammatory cytokines (such as TNF-α) in the hippocampus of male Wistar rats after scopolamine was intraperitoneally administered [73]. Sesame lignan (including sesamin and sesamol) has anti-inflammatory effects at a dose of 30 mg/kg in various neuropathological conditions, including brain ischemia [72], seizures [74], diabetic retinopathy [61], and scopolamine induction (Yun et al., 2022 [53]). Of note, Yun et al. [53] investigated the effect of sesame lignan on cholinergic disorders, neuronal inflammation, and cognitive deficits in scopolamine-induced mice, and reported that the mRNA expression of IL-6 and TNF-α was significantly upregulated by scopolamine, but sesamol treatment could downregulate the mRNA expression of these inflammatory cytokines. Scopolamine induces NF-κB and ERK phosphorylation and upregulates the expression of inflammatory cytokines, leading to neuronal inflammation [75]. However, lignan derived from sesame as a dietary supplement downregulates the expression of inflammatory cytokines by inhibiting microglial over-activation [76]. Microglia are the primary immune cells that produce inflammatory cytokines. Therefore, the influence of sesame extract and oil from the lignan-rich ‘Milyang 74’ cultivar on neuronal inflammation in scopolamine-treated mice was reflected by examining microglia activation and the expression of inflammatory cytokines by lignans such as sesamin, sesamolin, and sesaminol derived from sesame. Sesame extract oil from the lignan-rich cultivar ‘Milyang 74’ had a more enhanced preventive effect on scopolamine-induced neuronal inflammation.

## 4. Conclusions

The current study suggests the difference of in vitro/in vivo cognitive function improvement between sesame extract and oil due to lignan variation, in parallel with our previous study on development of lignan-rich ‘Milyang 74 (KACC 88003BP)’ cultivars, was artificially bred using ‘Gyeongbuk 22’, which contains a high lignan content and short stem length, and ‘YCS71’, which has a high lignan content and fast maturity as parental lines. According to screening results of functional compounds including individual lignan and in vitro antioxidant and enzyme inhibitory activities, we selected the ‘Milyang 74’ cultivar with high lignan content and effective inhibitory capacity regarding acetylcholinesterase to evaluate anti-neurotoxicity in amyloid-β-induced SH-SY5Y cells, and produces an in vivo nootropic effect in scopolamine-induced memory impaired mice. In the amyloid-β-induced SH-SY5Y cell model, sesame extracts from the lignan-rich ‘M74’ cultivar improve cell viability more in the presence of Aβ, and reduced ROS level from oxidative stress, and suppressed lipid peroxidation and intracellular antioxidant enzymes than the ‘Goenback’ control cultivar. Furthermore, in the scopolamine-induced memory impaired mice model, M74 extract (250 and 500 mg/kg) and oil (1 and 2 mL/kg) pretreatment improved learning and memory deficits in mice (as measured by the passive avoidance test), inhibited AChE, and enhanced acetylcholine (Ach) levels. Immunohistochemistry and Western blot results showed that the M74 extract and oil reversed the scopolamine-induced increase in APP, BACE-1, and presenilin expression in the amyloid cascade and decreased BDNF and NGF expression in neuronal regeneration. As a result, we concluded that lignan-rich cultivars with high amounts of sesamin, sesamolin, and sesamol have higher regulation capacity of cholinergic deficit characteristics and protein expression related to amyloid-β accumulation, neuronal regeneration, and neuro-inflammation lead to benefits in cognition and memory function.

## Figures and Tables

**Table 1:** Effect of lignan-rich ‘Milyang 74’ sesame cultivar on lipid peroxidation and antioxidant enzyme activities in amyloid-β-induced SH-SY5Y cell deaths compared to the ‘Goenback’ and ‘Daheuk’ control cultivars.

Sample	^1^ MDA	^2^ GR	^3^ GPX	^4^ CAT	^5^ SOD
Control	0.6 ± 0.02	10.32 ± 1.21	42.92 ± 1.97	5.26 ± 0.84	1.3 ± 0.26
Aβ	1.15 ± 0.11 ^###^	29.92 ± 1.37 ^###^	92.49 ± 2.52 ^###^	23.92 ± 2.66 ^###^	14.46 ± 2.65 ^###^
Aβ + DH 100	0.79 ± 0.01 ^a^	18.69 ± 1.97 ^a^	63.82 ± 3.51 ^a^	18.22 ± 2.00 ^a^	4.54 ± 0.88 ^a^
Aβ + GB 100	0.85 ± 0.05 ^a^	17.47 ± 0.98 ^a^	54.71 ± 3.50 ^b^	14.71 ± 0.74 ^b^	3.48 ± 0.47 ^a^
Aβ + M74 100	0.68 ± 0.03 ^b^	13.36 ± 0.86 ^b^	39.36 ± 1.56 ^c^	11.29 ± 1.07 ^c^	2.17 ± 0.18 ^b^

Values are means ± SD of 3 replicates. ^###^ *p* < 0.001 significant difference compared to control. Different small letters in the same items indicate a significant difference (*p* < 0.05) among different varieties. ^1^ Lipid peroxidation of malonaldehyde (MDA, nmol mg protein) and antioxidant enzyme activity of ^2^ glutathione reductase (GR, µmol min mg^−1^ protein), ^3^ glutathione peroxidase (GPx, μmol min mg^−1^ protein), ^4^ catalase (CAT, μmol min mg^−1^ protein), and ^5^ superoxide dismutase (SOD, unit mg^−1^ protein) were evaluated in SH-SY5Y cells treated for 24 h with the sample and 25 μM amyloid Aβ_25-35_ fragment.

## Data Availability

Data is contained within the article or supplementary material.

## Supplementary Materials

The following supporting information can be downloaded at: https://www.mdpi.com/article/10.3390/antiox12051110/s1, Table S1: Total polyphenol content, total flavonoid content, and sesame lignan composition in different sesame varieties; Table S2: Antioxidant activities and enzyme inhibitory activities of different sesame varieties; Table S3: In vivo experimental design; Table S4: Body weight (g), tissue weight (g), and serum biochemistry analysis.
