# Supplementary material for: Lignan-Rich Sesame (Sesamum indicum L.) Cultivar Exhibits In Vitro Anti-Cholinesterase Activity, Anti-Neurotoxicity in Amyloid-β Induced SH-SY5Y Cells, and Produces an In Vivo Nootropic Effect in Scopolamine-Induced Memory Impaired Mice
Source: Antioxidants (Basel). 2023 May 17;12(5):1110. doi: 10.3390/antiox12051110 (PMC10215706; doi:10.3390/antiox12051110)
Supplement: Supplementary file 1 [file antioxidants-12-01110-s001.zip › antioxidants-2353698-supplementary.pdf]

Article

Lignan-rich sesame (*Sesamum indicum* L.) cultivar exhibits *in vitro* anti-cholinesterase activity, anti-neurotoxicity in amyloid-β induced SH-SY5Y cells, and produces an *in vivo* nootropic effect in scopolamine-induced memory impaired mice

Min Young Kim[1*], Sungup Kim[1], Jeongeun Lee[1], Jung In Kim[1], Eunyoung Oh[1], Sang Woo Kim[1], Eunsoo Lee[1], Kwang Soo Cho[1], Myoung Hee Lee[1]

Supplementary Materials

Supplementary Table S1. Total polyphenol content, total flavonoid content, and sesame lignan composition in different sesame varieties.

Supplementary Table S2. Antioxidant activities and enzyme inhibitory activities of different sesame varieties.

Supplementary Table S3. *In vivo* experimental design.

Supplementary Table S4. Body weight (g), tissue weight (g), and serum biochemistry analysis.

Table S1. Total polyphenol content, total flavonoid content, sesame liganan composition of different sesame varieties.

Varieties	[1)]TPC (mg GAE/g sample)	[2)]TFC (mg CE/g Sample)	Lignan content(mg/g sample)					
			Sesamin	Sesamolin	Sesaminol	Sesaminol-diglucoside	Sesaminol-triglucoside	Total
Goenback	1.48±0.03^{d}	0.59±0.02^{e}	2.33±0.07^{e}	1.69±0.01^{e}	0.02±0.00^{h}	0.12±0.00^{g}	0.82±0.01^{f}	4.98±0.08^{fg}
Ansan	1.28±0.03^{f}	0.79±0.01^{d}	4.65±0.10^{c}	2.24±0.04^{c}	0.04±0.00^{b}	0.34±0.01^{b}	2.03±0.07^{c}	9.30±0.13^{d}
Koppom	1.41±0.02^{e}	0.76±0.02^{d}	5.30±0.07^{b}	2.79±0.03^{b}	0.04±0.00^{c}	0.31±0.00^{c}	1.90±0.03^{d}	10.34±0.09^{c}
Daheuk	0.85±0.04^{h}	0.36±0.01^{f}	1.25±0.07^{f}	1.19±0.06^{g}	0.02±0.00^{g}	0.19±0.01^{e}	1.24±0.02^{e}	3.90±0.10^{g}
Miryang 68	1.72±0.02^{c}	1.67±0.17^{a}	2.65±0.13d^{e}	1.98±0.03^{d}	0.02±0.00^{f}	0.13±0.00^{g}	0.65±0.01^{g}	5.44±0.15^{f}
Miryang 69	1.00±0.03^{g}	0.58±0.01^{e}	2.83±0.10^{d}	1.50±0.02^{f}	0.03±0.00^{d}	0.21±0.02^{e}	0.90±0.04^{f}	5.47±0.10^{f}
Miryang 70	0.76±0.01^{i}	0.32±0.02^{f}	2.46±0.07^{e}	1.43±0.01^{f}	0.02±0.00^{e}	0.18±0.01^{f}	1.27±0.04^{e}	5.35±0.10^{f}
Miryang 72	0.66±0.01^{j}	0.17±0.02^{g}	2.84±0.10^{d}	1.68±0.03^{e}	0.03±0.00^{e}	0.27±0.00^{d}	2.56±0.00^{b}	7.38±0.13^{e}
Miryang 73	2.39±0.04^{a}	1.04±0.05^{c}	10.25±0.51^{a}	3.58±0.19^{a}	0.04±0.00^{b}	0.35±0.01^{b}	2.61±0.09^{b}	16.83±0.61^{b}
Miryang 74	2.31±0.03^{b}	1.22±0.04^{b}	10.08±0.14^{a}	3.47±0.16^{a}	0.06±0.00^{a}	0.59±0.01^{a}	3.50±0.07^{a}	17.71±0.32^{a}

Values are mean ± SD of three replicates. Different small letters in the same items indicate a significant difference ($p < 0.05$) among varieties.

[1)]Total polyphenol content(mg gallic acid equivalent/g sample)

[2)]Total flavonoid content(mg catechin equivalent/g sample)

Table S2. Antioxidant activities and Enzyme inhibitory activities of different sesame varieties.

varieties	Antioxidant activities(mg TE/g sample)		Enzyme inhibitory activities(%)			
	[1)]ABTS	[2)]DPPH	[3)]AChE (0.4 mg/mL)	[4)]BChE (2 mg/mL)	[5)]ACE (10 mg/mL)	[6)]AG (2.5 mg/mL)
Goenback	3.55±0.05[c]	2.17±0.31[c]	32.49±1.21[g]	16.40±0.03[f]	25.33±7.90[ab]	53.56±3.10[e]
Ansan	2.54±0.07[f]	1.94±0.31[c]	37.17±0.98[f]	12.90±0.06[g]	18.62±0.91[b]	84.38±1.18[a]
Koppom	3.05±0.09[d]	2.06±0.25[c]	32.47±2.48[g]	14.60±0.18[h]	18.06±4.48[b]	82.18±1.43[ab]
Daheuk	2.68±0.02[e]	2.50±0.15[b]	30.47±3.07[h]	20.80±0.34[e]	30.24±5.43[a]	53.05±2.83[e]
Miryang 68	3.93±0.03[b]	2.58±0.20[b]	37.15±1.26[f]	26.70±0.16[d]	18.41±2.97[b]	56.12±2.44[e]
Miryang 69	2.20±0.05[g]	1.63±0.07[d]	43.57±1.79[c]	21.16±0.70[e]	29.67±4.06[a]	60.32±2.20[d]
Miryang 70	1.85±0.10[h]	1.29±0.04[e]	40.12±2.02[e]	27.58±0.55[d]	28.27±1.92[a]	78.33±2.12[b]
Miryang 72	1.61±0.10[i]	1.15±0.04[e]	41.78±0.90[d]	32.19±0.64[c]	27.75±8.71[a]	79.25±3.18[b]
Miryang 73	5.19±0.07[a]	2.98±0.09[a]	61.49±2.56[b]	38.70±0.97[a]	26.31±0.31[ab]	45.84±3.50[f]
Miryang 74	5.28±0.08[a]	2.98±0.08[a]	66.17±3.13[a]	36.40±0.11[b]	29.11±1.08[a]	67.63±2.54[c]

Values are mean ± SD of three replicates. Different small letters in the same items indicate a significant difference ($p < 0.05$) among varieties.

[1)]ABTS radical scavenging activity(mg trolox equivalent/g sample)

[2)]DPPH radical scavenging activity(mg trolox equivalent/g sample)

[3)]Acetylcholinesterase inhibitory activity(%)

[4)]Butylcholinesterase inhibitory activity(%)

[5)]Angiotensin converting enzyme inhibitory activity(%)

6)α-glucosidase inhibitory activity(%)

Table S3. *In vivo* Experimental design

No.	Group	Mice	Memory Impairment (Scopolamine, 2 mg/kg)	Sample concentration
1	N	7	-	0.9 % NaCl
2	C	7	+	0.9 % NaCl
3	P	7	+	0.75 mg/kg
4	GBE	7	+	250 mg/kg
5		7	+	500 mg/kg
6	GBO	7	+	1 mL/kg
7		7	+	2 mL/kg
8	M7**4**E	7	+	250 mg/kg
9		7	+	500 mg/kg
10	M74O	7	+	1 mL/kg
11		7	+	2 mL/kg

N: Normal, C: Control, P: Donepezil, GBE: Goenbaek extract, GBO: Goenbeak oil, M74E: M74 extract, M74O: M74 Oil

Table S4. Body weight(g), Tissue weight(g), serum biochemistry analysis

Group		Body weight (g)		Tissue weight(g)				Enzyme activities in serum(U/L)	
		Initial	4 week	Liver	Kidney	Spleen	Brain	GOT	GPT
N		29.52 ± 1.08$^{n.s}$	37.16 ± 1.58	2.26 ± 0.19	0.65 ± 0.08	0.14 ± 0.02$^{n.s}$	0.48 ± 0.03$^{n.s}$	67.26 ± 8.03†	37.58 ± 7.04$^{\#}$
C		29.54 ± 0.84	35.59 ± 1.31	2.14 ± 0.26	0.66 ± 0.09	0.12 ± 0.01	0.48 ± 0.03	101.81 ± 22.80	52.73 ± 10.16
P		29.50 ± 1.30	33.60 ± 0.95	2.08 ± 0.09	0.65 ± 0.06	0.11 ± 0.01	0.47 ± 0.03	70.59 ± 16.02†	35.94 ± 7.30$^{\#}$
GBE	250 mg/kg	29.61 ± 1.08	33.57 ± 1.40	2.16 ± 0.18	0.64 ± 0.08*	0.14 ± 0.02	0.48 ± 0.08	60.86 ± 2.45†	36.67 ± 1.91$^{\#}$
	500 mg/kg	29.53 ± 1.23	33.57 ± 2.27	2.18 ± 0.18	0.68 ± 0.04	0.11 ± 0.01	0.49 ± 0.03	69.99 ± 6.20†	37.06 ± 3.72$^{\#}$
GBO	1 mL/kg	29.46 ± 1.23	33.97 ± 1.50	1.96 ± 0.21	0.64 ± 0.05	0.12 ± 0.01	0.48 ± 0.02	55.93 ± 10.67†	32.33 ± 7.84†
	2 mL/kg	29.49 ± 0.75	33.35 ± 0.98*	1.88 ± 0.19*	0.62 ± 0.09	0.11 ± 0.02	0.49 ± 0.02	60.72 ± 8.68†	25.80 ± 6.85†
M74E	250 mg/kg	29.46 ± 0.97	35.44 ± 2.21	2.19 ± 0.18	0.66 ± 0.07	0.13 ± 0.02	0.48 ± 0.03	66.28 ± 9.44†	38.61 ± 9.99$^{\#}$
	500 mg/kg	29.64 ± 0.95	34.44 ± 1.65	2.21 ± 0.26	0.72 ± 0.06	0.12 ± 0.02	0.49 ± 0.01	54.09 ± 8.76†	28.89 ± 2.67†
M74O	1 mL/kg	29.64 ± 0.91	33.68 ± 1.79*	2.01 ± 0.21	0.64 ± 0.10	0.12 ± 0.02	0.47 ± 0.02	62.57 ± 12.64†	31.59 ± 14.83†
	2 mL/kg	29.48 ± 0.98	33.33 ± 1.62*	1.88 ± 0.17*	0.60 ± 0.05	0.12 ± 0.02	0.47 ± 0.03	66.95 ± 7.09†	29.65 ± 6.16†
